# Acute Viral Hepatitis Due to Co-infection With Hepatitis A and Hepatitis B in an Intravenous Drug User

**DOI:** 10.7759/cureus.37179

**Published:** 2023-04-05

**Authors:** Shaina Logemann, Richard Blinkhorn

**Affiliations:** 1 Internal Medicine, MetroHealth Medical Center, Cleveland, USA; 2 Internal Medicine, Case Western Reserve University, Cleveland, USA

**Keywords:** outcome of co-infection, acute viral hepatitis, hav, hepatitis b(hbv), injection drug use

## Abstract

Injection drug users are at high risk of contracting human immunodeficiency virus (HIV), hepatitis B virus (HBV), and hepatitis C virus (HCV) due to parenteral exposure. Hepatitis A virus (HAV) is classically thought to be transmitted through the fecal-oral route, but injection drug use is increasingly recognized as a risk factor. It is well documented that there is a high prevalence of total antibodies to HAV in injection drug users, although there is limited data about the prevalence of acute HAV in injection drug users. Acute viral hepatitis is most often due to HAV, HBV, or hepatitis E virus (HEV), and it is rare to have acute co-infection with these viruses. We report a case of acute viral hepatitis due to co-infection with both HAV and HBV in an injection drug user.

## Introduction

It is well recognized that injection drug users are at significant risk of contracting viral infections such as human immunodeficiency virus (HIV), hepatitis B virus (HBV), and hepatitis C virus (HCV), due to parenteral exposure. Classically, the method of transmission of hepatitis A virus (HAV) has been via the fecal-oral route due to poor sanitation and/or hygiene, however, injection drug users are also at risk for contracting HAV. Studies have shown that injection drug users are exposed to HAV through needle sharing, poor hygiene, and absence of hand washing leading to contamination of drug-mixing containers, and sexual contact with people infected with hepatitis [1-5].

Co-infection with HIV, HBV, and/or HCV has been reported in injection drug users [6-8], however, there is very limited data regarding co-infection with HAV. Seroprevalence studies have found antibodies to both HAV and HBV in injection drug users, but these studies either tested for total antibodies to HAV (rather than immunoglobulin M) or did not specify the type of antibody [9,10]. Another study found that while over half of injection drug users tested positive for anti-HAV total antibody, none of them tested positive for anti-HAV immunoglobulin M (IgM), indicating that none had acute infection with HAV [11]. There have only been two other case reports of acute co-infection with HAV and HBV in general [12,13].

Typically, acute HAV infection is a self-limiting disease and rarely causes fulminant hepatic failure. Acute HBV infection can be subclinical or cause similar symptoms to that of acute HAV infection. Additionally, it is understood that superinfection with acute HAV in patients with chronic HBV or HCV leads to increased severity of hepatic damage due to underlying chronic hepatocellular injury, resulting in possible fulminant liver failure and even death [14]. However, acute co-infection with multiple hepatitis viruses is not well documented, and thus the risk of acute liver failure secondary to co-infection is unknown. We report a case of acute co-infection with HAV and HBV in an injection drug user without other known risk factors for acquiring acute hepatitis.

## Case presentation

A 36-year-old female with a history of daily intravenous methamphetamine use and untreated HCV presented with epigastric and right upper quadrant abdominal pain, fevers, chills, myalgias, dark urine, nausea, and decreased appetite for one week. She reported sharing needles with her partner while using drugs; she reported that her partner had been jaundiced in the past, but she was not sure if he had been diagnosed with liver disease. She denied the use of alcohol or medications that could affect hepatic function. She was not obese and had no history of hyperlipidemia or diabetes. She denied a family history of liver disease. She denied recent travel, daycare exposure, and shellfish intake. She had been told she had HCV in the past when she attempted to donate plasma, but she had never received treatment. She was unsure of her vaccination status against HAV and HBV.

On examination, she was afebrile and hemodynamically stable. She had diffuse abdominal tenderness to light palpation but no rebound tenderness or organomegaly. She had sublingual icterus without scleral icterus. There were no stigmata of chronic liver disease. She did not have asterixis or altered mental status. Laboratory testing revealed a white blood cell count (WBC) of 2.9 (reference range 4.5-11.5 K/uL) with 41.3% neutrophils, 38% lymphocytes, 16.4% monocytes, 2.9% eosinophils, and 1.4% basophils; aspartate aminotransferase (AST) of 2,700 (reference range 7-40 IU/L), alanine aminotransferase (ALT) of 3,281 (reference range 7-40 IU/L), total bilirubin of 2.3 (reference range 0.1-1.5 mg/dL), direct bilirubin of 1.5 (reference range 0.10-0.30 mg/dL), and international normalized ratio (INR) of 1.41 (reference range 0.90-1.10). Investigation for the cause of acute hepatitis revealed a negative acetaminophen level, positive anti-HAV IgM, positive HBV surface antigen, positive HBV envelope antigen, negative HBV surface antibody, negative HBV core antibody, positive HCV antibody, negative hepatitis delta antibody, and negative HIV. Her HBV viral load was 260,000 copies and her HCV viral load was undetectable. Right upper quadrant ultrasound showed cholelithiasis without cholecystitis and an unremarkable liver without signs of cirrhosis.

**Figure 1:**
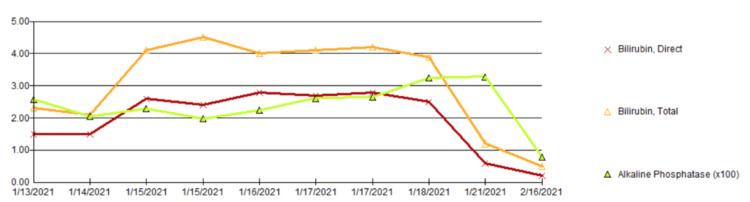
Bilirubin and Alkaline Phosphatase Trends

**Figure 2:**
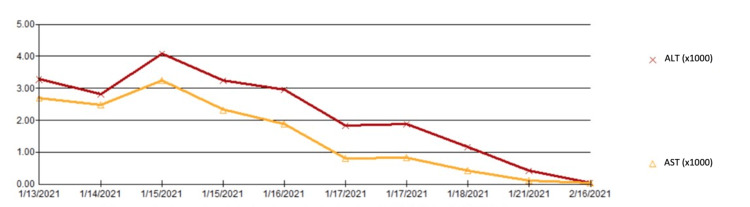
Alanine Aminotransferase (ALT) and Aspartate Aminotransferase (AST) Trends

She was treated with vitamin K with improvement in INR. Gastroenterology was consulted and did not recommend antiviral treatment for acute HBV. Liver function tests peaked at an ALT of 4,072 and AST of 3,252; her transaminases declined over the course of her stay (see Figure 1 and Figure 2). She was discharged in stable condition to a facility for drug rehabilitation. AST, ALT, and bilirubin had all normalized one month after discharge.

## Discussion

Intravenous drug users can be vulnerable to acute hepatitis from multiple hepatitis viruses, although acute co-infection is rare. In cases of acute hepatitis, patients should be tested for all hepatitis viruses, including HAV. Notably, there is limited data regarding co-infection with HAV, and most studies only documented the presence of total antibodies to HAV (indicating past exposure to the virus). Injection drug use has been increasingly noted as a risk factor for the acquisition of HAV. This case is unique in that this patient was diagnosed with acute hepatitis from both HAV and HBV, as evidenced by positive anti-HAV IgM, positive HBV surface antigen, positive HBV envelope antigen, and high HBV viral load. She had previously been diagnosed with HCV, however, her undetectable viral load indicated she had spontaneously cleared HCV in the past. Her only risk factor for acquiring acute hepatitis was injection drug use. She was not offered antiviral treatment for HBV as she was not in acute liver failure (as she did not experience encephalopathy and did not have a significant coagulopathy with INR >1.5). Superinfection with acute HAV in a patient with chronic HBV or HCV places the patient at higher risk of more severe liver injury and even fulminant liver failure requiring liver transplantation. Little is known about the risks or severity of acute co-infection with HAV and HBV. Fortunately, this patient did not suffer liver failure and her liver injury had completely resolved within a month after discharge from the hospital. She was referred to the liver clinic, but unfortunately, she did not follow up.

## Conclusions

Injection drug use has increasingly become an epidemic in recent years in the United States. Providers should thoroughly discuss the health risks of injection drug use with patients, including potential HIV, HBV, and HCV but also HAV. It is now known that injection drug users are particularly susceptible to acquiring HAV through multiple routes, including needle sharing, poor hygiene, and sexual contact. Providers should routinely screen even asymptomatic patients for HIV and all hepatitis viruses. Many organizations recommend vaccinating patients with chronic HCV against HAV and HBV; however, patients without HCV who use injection drugs should also be candidates for these vaccinations as a preventative measure. It is particularly important to keep all hepatitis viruses on the differential in cases of acute hepatitis. Finally, if a patient is diagnosed with acute co-infection with multiple hepatitis viruses, early consultation with a liver specialist is crucial. Consideration of antiviral treatment is necessary. It may be prudent to consider early initiation of antiviral treatment for HBV even in the absence of acute liver failure, as there is no established treatment for acute HAV.
